# An open-access dataset of odontocete acoustic signals from the Madeira archipelago

**DOI:** 10.1038/s41597-026-07675-5

**Published:** 2026-06-24

**Authors:** Laura Redaelli, Francesco Caruso, Manuel E. dos Santos, Filipe Alves, Ana Dinis

**Affiliations:** 1 MARE – Marine and Environmental Sciences Centre/ARNET – Aquatic Research Network, Regional Agency for the Development of Research, Technology and Innovation (ARDITI), Funchal, Madeira Portugal; 2 Faculty of Life Sciences, University of Madeira, Funchal, Portugal; 3 MARE – Marine and Environmental Sciences Centre/ISPA - Instituto Universitário, Lisbon, Portugal; 4 Department of Marine Animal Conservation and Public Engagement, Stazione Zoologica Anton Dohrn, Naples, Italy

## Abstract

Acoustic monitoring provides a powerful, non-invasive approach for studying marine ecosystems, offering insights into species presence, behaviour, and habitat use. Odontocetes rely heavily on sound for communication, navigation, and foraging, making their vocalisations key indicators of ecological and social processes. This study presents a curated, open-access dataset of odontocete acoustic recordings collected between 2022 and 2025 in the waters of the Madeira Archipelago, Eastern North Atlantic. The dataset helps to address a major geographic gap in publicly available bioacoustics resources from the Macaronesian region, a recognized biodiversity hotspot in the North Atlantic. Acoustic data were obtained using a SoundTrap 300HF hydrophone recording at a sampling rate of 288 kHz, during boat-based surveys of visually confirmed single-species sightings at close range. The collection includes annotated recordings from seven odontocete species, encompassing whistles, broadband pulsed calls and echolocation clicks. Each audio file is accompanied by detailed metadata that describes the species identity, group size, behaviour, and environmental context. This resource supports comparative bioacoustics research and the development of automated tools for passive acoustic monitoring.

## Background & Summary

Marine habitats cover most of the planet’s surface and host an extraordinary diversity of biological sounds produced by organisms, ranging from invertebrates to large marine mammals^1^. Because sound travels efficiently in water, acoustic signals provide reliable cues for detecting biological activity, characterizing habitats, and monitoring ecological change^2–4^. Among marine mammals, odontocetes are particularly dependent on sound for survival. They use a complex acoustic repertoire for communication, social coordination, navigation, and foraging, making vocal behaviour central to their ecology and evolution^5^.

Passive acoustic monitoring (PAM) has become a fundamental non-invasive method for studying marine mammal vocal behaviours and assessing species presence, distribution, and social or ecological dynamics across temporal and spatial scales^6,7^. Beyond ecological monitoring and soundscape characterization, PAM provides a unique window into vocal communication systems, enabling detailed analyses of call structure, repertoire composition, and behavioural context. Open-access, labelled acoustic datasets of odontocete sounds serve as critical resources for these investigations, allowing researchers to examine species- or population-specific repertoires, quantify vocal variability, and explore links between acoustic behaviour and ecological or social factors (e.g.^8–10^). Such datasets also underpin the development and validation of automated classification algorithms, which are increasingly used to analyse large-scale acoustic archives and detect species in real time^11–14^.

Despite growing global initiatives to make marine acoustic data publicly accessible, the geographic coverage of open datasets remains uneven. Tropical and subtropical regions of the Atlantic are particularly underrepresented, limiting comparative research on odontocete vocal behaviour across ocean basins. The Macaronesian region, which includes the archipelagos of the Azores, Madeira, Canary Islands, and Cabo Verde, is recognized as both a biodiversity hotspot and a key biogeographic transition zone in the Atlantic^15–18^. These archipelagos host a rich cetacean assemblage, including highly island-associated groups such as common bottlenose dolphins (*Tursiops truncatus*) and short-finned pilot whales (*Globicephala macrorhynchus*), as well as temporary visitors, such as small-sized delphinids and baleen whales, that use the area seasonally or as a migratory corridor^15,19–21^. Despite this exceptional importance, publicly available data from the region remains largely limited to species occurrence and biodiversity records^22–24^, with no open-access acoustic datasets for cetaceans currently available. This limitation is especially evident in the Madeira Archipelago, where peer-reviewed publications based on cetacean acoustic datasets are currently scarce^25^, restricting behavioural, ecological, and comparative bioacoustics research.

This paper introduces a curated, open-access dataset of odontocete acoustic recordings collected in the waters of the Madeira Archipelago. The dataset includes annotated recordings from seven odontocete species commonly occurring in the region: short-beaked common dolphin (*Delphinus delphis*), Risso’s dolphin (*Grampus griseus*), short-finned pilot whale (*Globicephala macrorhynchus*), sperm whale (*Physeter macrocephalus*), rough-toothed dolphin (*Steno bredanensis*), Atlantic spotted dolphin (*Stenella frontalis*), and common bottlenose dolphin (*Tursiops truncatus*). The recordings encompass a variety of vocalisation types, including whistles, pulsed calls, and echolocation clicks. Each sound file is accompanied by metadata describing species identification, estimated group size, behavioural state, and environmental variables, such as recording location, date, and sea state.

The dataset is intended to support research on species identification, call classification, and comparative analyses of odontocete vocal behaviour. It may also assist in training and testing automated detection and classification algorithms and contribute to regional and global efforts to document marine mammal acoustic diversity.

## Methods

### Data collection

**Fig. 1:**
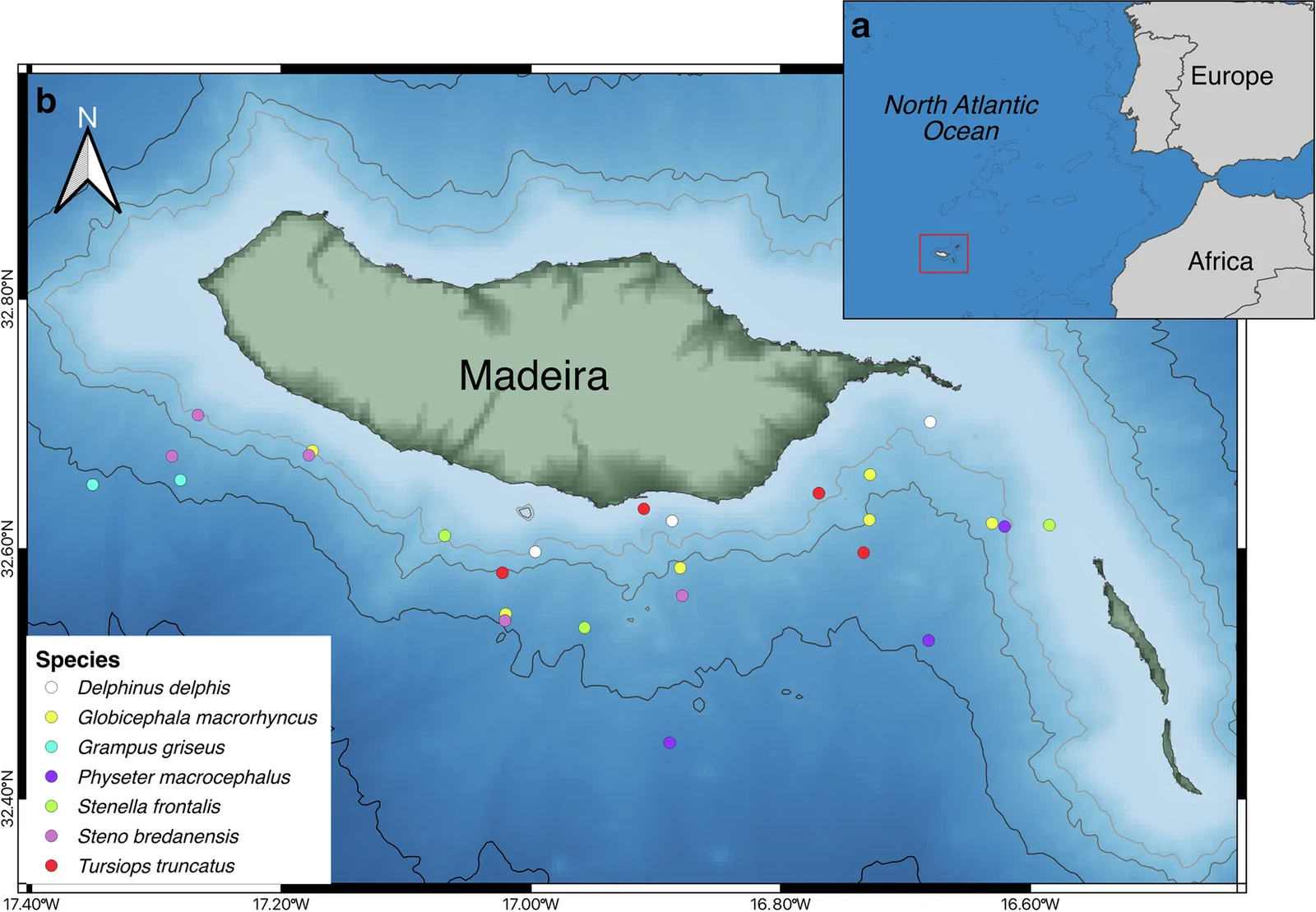
Study area. (**a**) location of the Madeira Archipelago in the Eastern North Atlantic. (**b**) detailed map of the study area, showing the positions of all odontocete sightings for which acoustic recordings are available. Each colored point represents a sighting, with colors indicating different species.

Underwater acoustic recordings of free-ranging odontocetes were collected between 2022 and 2025 off the south coast of Madeira Island, Portugal (Eastern North Atlantic; Fig. 1), during dedicated boat-based surveys. Each recording event corresponded to a visually confirmed encounter with an odontocete group.

Species identification was conducted in the field by experienced observers, and later validated through photographs taken with digital cameras (Canon EOS 7D Mark II; 400 mm lenses). Only single-species sightings were included, defined as encounters where all individuals were visually identified as belonging to one species and no other cetaceans were observed in the vicinity.

Acoustic data were collected using a SoundTrap 300HF recorder (Ocean Instruments Inc., New Zealand) with a sampling rate of 288 kHz in continuous recording mode. This instrument presents a linear frequency range of 20 Hz – 150 kHz ± 3 dB, and sensitivity of −201 dB re V/µPa (high gain setting with a maximum sound pressure level of 174.7 dB re 1 µPa peak to peak before clipping). The hydrophone was deployed from a stationary vessel at depths between 5 and 30 m, depending on sea conditions and the focal species. The recorder was attached to a nylon line equipped with a swivel to reduce cable noise. Deployments were conducted only when the target animals were within approximately 300 m of the vessel. To minimize self-noise and animal disturbance, the vessel’s engine was turned off during recordings. When repositioning was required due to animal movement, the hydrophone was retrieved, the recording manually paused, and redeployed once a suitable position was reached.

### File management and preparation

All recordings were downloaded from the recorder using the manufacturer’s SoundTrap Host software. Raw audio files were saved in uncompressed WAV format at the original sampling rate of 288 kHz and 16-bit resolution. Each recording session was named using a standardized convention (YYYYMMDD_SightingID_SpeciesCode.wav) to ensure traceability between the recordings and the corresponding field metadata.

All audio files were visually and aurally inspected in Raven Pro 1.6.5^26^ to verify signal quality and confirm the presence of odontocete sounds. Files affected by excessive handling noise, clipping, or hardware malfunction were excluded. To preserve the integrity of the dataset, no amplitude normalization, filtering, or noise reduction was applied to the archived recordings.

### Metadata integration

Metadata recorded during field surveys were digitized and cross-checked for completeness and internal consistency before being compiled into a single metadata table. Each entry corresponds to one sighting event and includes temporal, geographic, and contextual information linking the field observations to the corresponding audio files.

## Data Records

The dataset presented in this study is open-access and accessible through Zenodo^27^.

Within the Zenodo repository, there are two ZIP folders containing uncompressed audio files in WAV format, and a comma-separated values (CSV) file containing the associated metadata.

### Dataset (WAV files)

This ZIP folder contains 35 acoustic recordings of odontocete vocalisations of varying durations, organised into subfolders by species using specific name codes. Each species folder includes between three and six recordings of variable durations (min-max (mm:ss.0): 1:15.6–15:45.0).

### Metadata (CSV file)

The file *Metadata.csv* provides detailed information for each recording. The following variables are included:

- **Species:** Scientific name of the species.
- **Common name:** English common name of the species.
- **Species code:** Abbreviation assigned to each species.
- **WAV file name:** Identifier corresponding to the associated WAV file. It is composed of the date of the recording (in format YYYYMMDD), the sighting ID and the species code.
- **Duration (mm:ss.0):** Total duration of the recording.
- **Date:** Date of the sighting.
- **Latitude:** Latitude of the initial sighting position.
- **Longitude:** Longitude of the initial sighting position.
- **Recording start** (hh:mm:ss): time of the start of the recording.
- **Recording end** (hh:mm:ss): time of the end of the recording.
- **Sea state:** Sea surface condition based on the Beaufort scale.
- **Hydrophone depth** (meters): Depth of the recorder.
- **Sampling rate** (kHz): hydrophone’s sampling rate.
- **Group size:** Estimated number of individuals observed.
- **Calves:** Number of calves observed in the group.
- **Group behaviour:** Predominant behavioural category observed during the sighting (travelling, resting, socialising, feeding, erratic) following the definitions in Alves *et al*.^15^. Behaviour was assigned based on the activity in which the majority of the group was engaged. When a substantial portion of the group simultaneously displayed a different behaviour, or when the predominant behaviour changed during the recording, both categories were reported.
- **Boat presence:** Presence or absence of other vessels besides the research vessel.
- **Boat type:** Type of vessel present (WW: whale watching; FV: fishing vessel) besides the research vessel.
- **Boat activity:** Activity of the vessels present in the area during the recordings (IDL: idling; TrF: travelling fast; TrS: travelling slow). Since the research vessel remained stationary with the engine turned off throughout all recording sessions, this category refers exclusively to the activity of other vessels in the vicinity.
- **Vocalisation types:** Vocalisation categories identified in each recording.
- **Notes:** Additional remarks or contextual information relevant to data interpretation.

### Technical Validation (WAV, PDF and PNG files)

The *Technical Validation* ZIP folder provides all files and information used to check the quality of the data. It contains:

- **Audio Files 1-min:** folder containing 1-min recordings for each species (throughout the text, these are referred to as “snippets”).
- **Spectrogram-PSD:** folder containing PNG files displaying spectrogram and Power Spectral Density (PSD) for each species using the 1-min snippets.
- **Soundtrap_CalibrationSheet**: PDF file reporting hydrophone manufacturer information on the calibration.
- **Calibration files:** folder containing the calibration tone files (WAV) corresponding to each recording, enabling users to verify and apply the appropriate calibration parameters if required.

See the following section for details.

## Technical Validation

The recorder was factory-calibrated, and the calibration documentation is included in the accompanying metadata. In accordance with the manufacturer’s specifications, each recording begins with a series of calibration tones used to verify system sensitivity and performance. After applying the calibration parameters, the sound pressure level (SPL) of the 1 kHz tone corresponds to the reference value provided in the calibration sheet (133.7 dB re 1 μPa). Calibration tones were retained in all recordings to allow post hoc verification of system accuracy.

The sampling frequency of 288 kHz allows representation of signals up to half that value due to the Nyquist frequency, enabling a recording bandwidth of 144 kHz and thus capturing all major acoustic characteristics of the recorded species.

To evaluate the quality and detectability of biological signals within the dataset, we extracted a representative one-minute snippet for each species that was visually inspected to confirm the presence, structure, and clarity of species-typical vocalisations. PSD analyses were then conducted using PAMGuide^28^, a MATLAB-based^29^ toolbox (version R2022b) widely used for standardized, calibrated underwater acoustic measurements. PAMGuide computes spectral metrics following conventional passive-acoustic monitoring procedures and incorporates the system calibration, allowing for fully comparable, absolute sound pressure levels across datasets.

For each snippet, we generated PSD curves using a Hann window (4112 samples), 50% overlap, and a frequency range of 20–144,000 Hz, with calibration applied using the end-to-end system sensitivity (−174.7 dB re 1 V µPa^−1^). From these PSDs, we extracted root-mean-square (RMS) sound pressure levels and compared them with PSD curves computed from baseline ambient-noise recordings. Ambient-noise data were collected at similar deployment depths used during species encounters (7 m or 30 m), under low sea-state conditions (Beaufort 1), and in areas free of vessel presence or other identifiable sources of noise, ensuring a reliable environmental baseline for comparison.

**Fig. 2:**
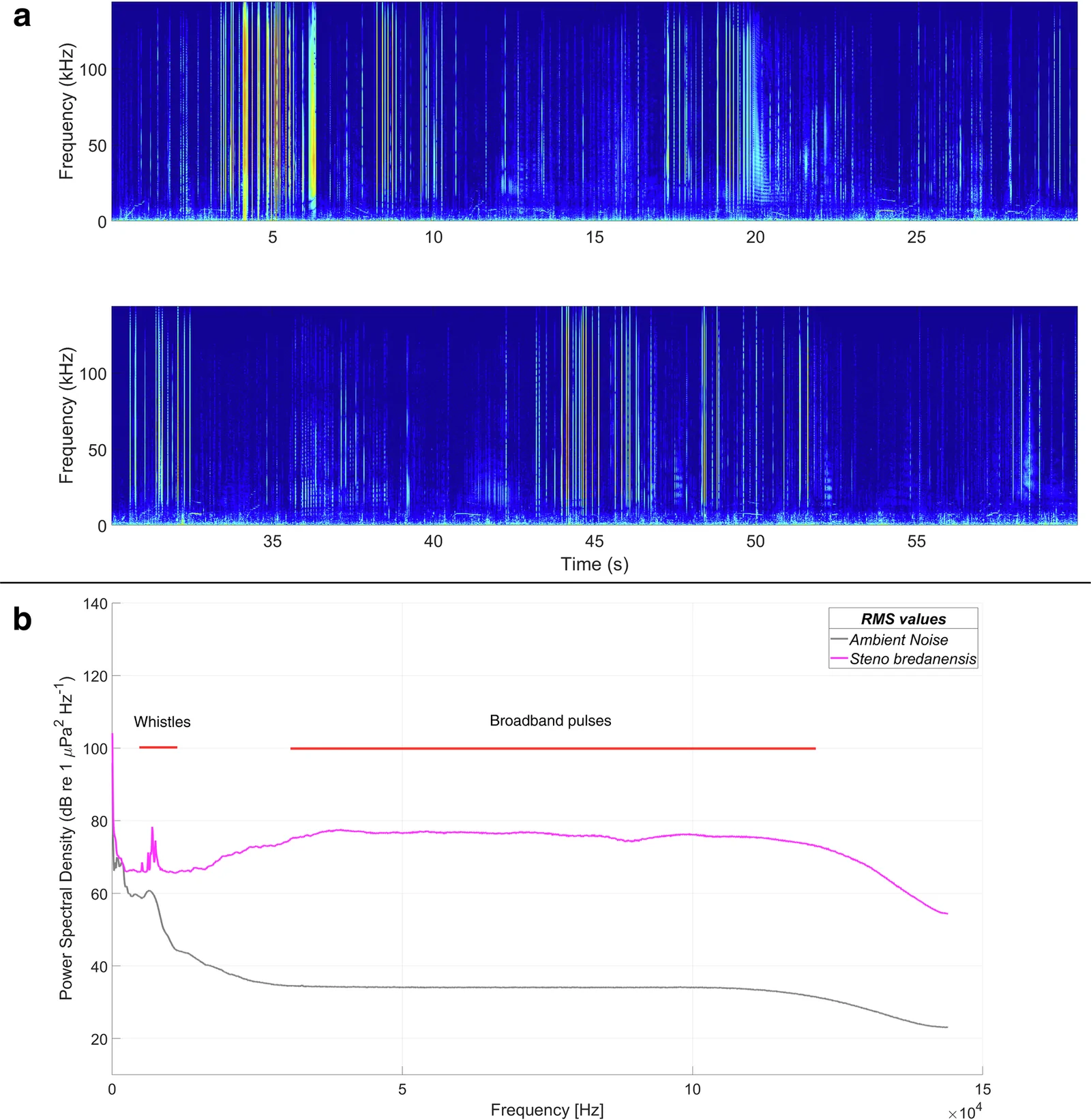
Technical validation example. (**a**) spectrograms of a 1-min recording (shown in two consecutive 30-s segments) illustrating whistles and broadband pulsed signals from rough-toothed dolphins *(Steno bredanensis)*. Spectrograms were computed in MATLAB using a Hann window, 90% overlap, and a 4096-point FFT. The signal was analysed at the native sampling rate (288 kHz), providing a bandwidth of 144 kHz. (**b**) Root-mean-square (RMS) values of the sound pressure levels (Power Spectral Density, dB re 1 µPa^2^ Hz^−1^) of the same file in comparison with the baseline ambient-noise recording. PSDs were computed using a Hann window of 4112 samples, 50% overlap, and a frequency range from 20 to 144,000 Hz. Acoustic pressure levels were calibrated using the end-to-end system sensitivity (−174.7 dB re 1 V µPa^−1^).

For each species, PSD curves from the annotated recordings consistently showed distinct energy peaks at frequencies corresponding to the dominant call types. These peaks were highlighted in the figures to demonstrate the features responsible for the elevated RMS values relative to baseline noise. In all cases, odontocete signals exceeded ambient noise levels by a clear margin, confirming a strong and well-defined signal-to-noise ratio. Figure 2 shows an example of this workflow illustrating a 1-min recording with whistles and broadband pulsed sounds from rough-toothed dolphins (*Steno bredanensis*). The equivalent plots for all the species are provided in the accompanying data repository. Collectively, all these technical checks demonstrate that the dataset provides high-quality, well-characterized acoustic recordings.

**Fig. 3:**
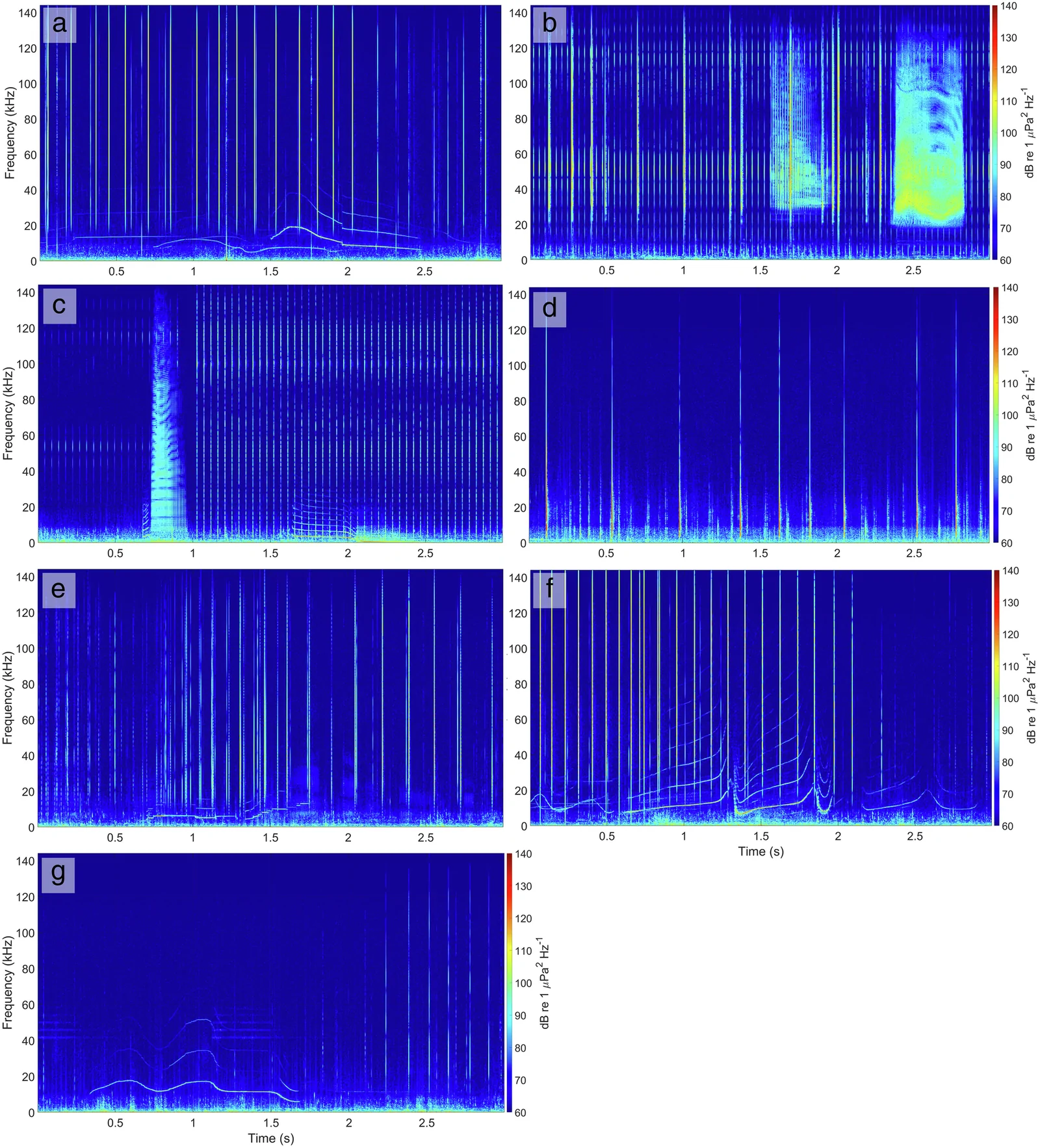
Representative calibrated spectrograms of acoustic signals from seven odontocete species. Each panel displays a 3-second snippet illustrating the varied vocal repertoires of seven species. (**a**) *Delphinus delphis*, whistles and echolocation clicks; (**b**) *Grampus griseus*, echolocation clicks and broadband pulsed calls; (**c**) *Globicephala macrorhynchus*, tonal and pulsed calls, echolocation clicks; (**d**) *Physeter macrocephalus*, regular clicks; (**e**) *Steno bredanensis*, whistles, broadband pulsed calls and echolocation clicks; (**f**) *Stenella frontalis*, whistles and echolocation clicks; (**g**) *Tursiops truncatus*, whistle and echolocation clicks. Spectrograms were computed in MATLAB using a Hann window, 90% overlap, and a 4096-point FFT.

To further validate the acoustic data, we provide representative 3-second calibrated spectrograms illustrating the variety of signals produced by each recorded species (Fig. 3).

## Data Availability

The data presented in this study are accessible through Zenodo^27^ (https://doi.org/10.5281/zenodo.17952229). Detailed description of the accessible files can be read in the “Data records” section of this article.

## Code availability

All analyses were carried out using MATLAB software (version R2022b) and PAMGuide (version registered on 27/01/2015). The custom code to plot calibrated spectrograms is provided in the technical validation folder at the link provided.
